# Immune Profiling of Cord Blood From Preterm and Term Infants Reveals Distinct Differences in Pro-Inflammatory Responses

**DOI:** 10.3389/fimmu.2021.777927

**Published:** 2021-11-01

**Authors:** Jeremy Anderson, Cao Minh Thang, Le Quang Thanh, Vo Thi Trang Dai, Van Thanh Phan, Bui Thi Hong Nhu, Do Ngoc Xuan Trang, Phan Thi Phuong Trinh, Thuong Vu Nguyen, Nguyen Trong Toan, Christopher M. Harpur, Kim Mulholland, Daniel G. Pellicci, Lien Anh Ha Do, Paul V. Licciardi

**Affiliations:** ^1^ Infection and Immunity, Murdoch Children’s Research Institute, Melbourne, VIC, Australia; ^2^ Department of Paediatrics, University of Melbourne, Melbourne, VIC, Australia; ^3^ Department of Microbiology and Immunology, Pasteur Institute of Ho Chi Minh City, Ho Chi Minh, Vietnam; ^4^ Tu Du Hospital, Ho Chi Minh, Vietnam; ^5^ Department of Labour Delivery, Tu Du Hospital, Ho Chi Minh, Vietnam; ^6^ Pasteur Institute of Ho Chi Minh City, Ho Chi Minh, Vietnam; ^7^ Clinical Research Centre, Pasteur Institute of Ho Chi Minh City, Ho Chi Minh, Vietnam; ^8^ Epidemiology and Public Health, London School of Hygiene and Tropical Medicine, London, United Kingdom; ^9^ Department of Microbiology and Immunology, University of Melbourne, Melbourne, VIC, Australia

**Keywords:** preterm, infant, immune profile, infection, inflammation

## Abstract

**Background:**

Preterm infants are highly vulnerable to infectious disease. While many factors are likely to contribute to this enhanced susceptibility, the immature nature of the preterm immune system is postulated as one key factor.

**Methods:**

In our study, we used high-dimensional flow cytometry and cytokine assays to characterise the immune profiles in 25 preterm (range: 30.4-34.1 weeks gestational age) and 25 term infant (range: 37-40 weeks gestational age) cord blood samples.

**Results:**

We found that preterm infants exhibit reduced frequencies of monocytes, CD56^bright^ NK cells, CD8+ T-cells, γδ T-cells and an increased frequency of intermediate monocytes, CD4+ T-cells, central memory CD4+ and CD8+ T-cells, Tregs and transitional B-cells compared to term infants. Pro-inflammatory cytokines IL-1β, IL-6 and IL-17A were lower in preterm infants in addition to chemokines IL-8, eotaxin, MIP-1α and MIP-1β. However, IL-15 and MCP-1 were higher in preterm infants.

**Conclusion:**

Overall, we identify key differences in pro-inflammatory immune profiles between preterm and term infants. These findings may help to explain why preterm infants are more susceptible to infectious disease during early life and facilitate the development of targeted interventions to protect this highly vulnerable group.

## Introduction

Preterm infants born <37 weeks gestational age (wGA) are highly susceptible to severe infectious disease (1). Moderate preterm infants born between 30-34 wGA are more likely to be hospitalised for infectious disease when compared to term infants (1, 2). One of the major factors implicated in the susceptibility of preterm infants to infectious disease is their immune system status (3). This highlights the need to understand the immune profile of preterm infants, particularly those who are moderate preterm as these infants represent a large proportion of preterm birth, with only limited immunological data currently available (4).

The preterm infant immune response is considered immature compared to term infants (3), as indicated by studies demonstrating reduced immune cell frequencies, total numbers and functionality in preterms (5, 6). For example, lower frequencies of natural killer (NK) cells in very preterm infants were associated with increased late onset sepsis with *Pseudomonas aeruginosa, Klebsiella pneumoniae, Staphylococcus haemolyticus* and *Escherichia coli* at 10.5 days post-birth (7). Similarly, reduced frequencies of T-cells and NK cells have been associated with severe respiratory syncytial virus (RSV) disease (8).

Identifying differences in immune profiles between preterm and term infants could be crucial to understanding susceptibility to infectious disease. However, literature regarding an in-depth comparison between the immune cell profiles in preterm and term infants is limited. In our study, we compared the immune profiles in cord blood between preterm and term infants, using a combination of high-dimensional flow cytometry and multiplex cytokine analysis.

## Methods

### Sample Collection and Study Cohort

Cord blood samples were obtained from a cohort of 25 healthy moderate preterm (30.4-34.1 wGA) and 25 healthy term (37-40 wGA) infants from Tu Du Hospital, Ho Chi Minh City, Vietnam.

All infants in this study were born by normal vaginal delivery and were free of early onset neonatal infection. Cord blood was not collected from infants who had:

- Major or suspected major malformations, including congenital heart disease, genetic syndromes,
- Clinical evidence of chorioamnionitis,
- Rupture of membranes for more than 24 hours,
- Suspected or confirmed early onset sepsis.

Or if their mothers had:

- Autoimmune disease or immunodeficiency syndrome, or immunosuppressant or immunomodifying treatment for more than 3 months.
- Infection: human immunodeficiency virus, hepatitis B, hepatitis C, primary herpes simplex virus infection during current pregnancy.
- Physical, psychiatric, or complex social situation where the mother and baby may not be able to fully participate such as maternal alcohol or substance dependency, issues about child protection.

The cord blood samples were transported to the Pasteur Institute of Ho Chi Minh City, Vietnam for cord blood mononuclear cells (CBMCs) isolation by density grade ficoll separation, and serum separation within 4 hours of collection. CBMCs and sera were stored in liquid nitrogen and -80°C respectively, until shipment to the Murdoch Children’s Research Institute, Melbourne, Australia.

This study was approved by the Pasteur Institute Ho Chi Minh City Ethics Committee (Ethics approval: 213/QD-PAS) and RCH Human Research Ethics Committee (HREC; 56904).

### Study Reagents

RPMI-160, fetal bovine serum (FBS), L-glutamine and penicillin-streptomycin were purchased from Sigma-Aldrich, St. Louis, USA. Anti-mouse compensation beads were purchased from BD Bioscience, San Diego, CA, USA. All flow cytometry antibodies used, and their suppliers are indicated in **Supplementary Table 1**.

### Flow Cytometry

Cryopreserved CBMCs were thawed at 37°C then washed with 10ml R_10_ media (RPMI-1640 medium supplemented with 10% FBS, 2mM L-glutamine, 1000IU penicillin-streptomycin) and centrifuged at 400 x g for 5 minutes. CBMCs were washed with 5ml PBS and centrifuged at 400 x g for 5 minutes then blocked (50μl of 1% human FC-block and 10% normal rat serum in PBS) for 20 minutes on ice. CBMCs were then washed with 1ml FACS buffer (PBS supplemented with 2% FBS and 2mM EDTA) and stained with 50µl of antibody cocktail 1 or 2 (**Supplementary Table 1**) for 20 minutes on ice. CBMCs were then washed and resuspended in 100µl FACS buffer for acquisition using the Cytek Aurora. Compensation was performed at the time of acquisition using compensation beads. Data was analysed using Flowjo v10.7.1 software. Gating strategies are shown in **Supplementary Figures 1**, **2**.

### Multiplex Cytokine/Chemokine Assay

A commercial multiplex bead array kit (27-plex human cytokine assay; Bio-rad, New South Wales, Australia) was used to measure IL-1β, IL-1ra, IL-2, IL-4, IL-5, IL-6, IL-7, IL-8, IL-9, IL-10, IL-12(p70), IL-13, IL-15, IL-17A, eotaxin, FGF-basic, G-CSF, GM-CSF, MCP-1, IFN-γ, TNF-α, IP-10, RANTES, MIP-1α, MIP-1β, PDGF and VEGF from serum according to manufacturer’s instructions. Results were analysed on a Luminex 200 instrument (Luminex, Texas, USA) fitted with the Bio-Plex Manager Version 6 software and results were reported in pg/ml.

### Statistical Analysis

Flow cytometry data was presented as a boxplot (min-max whiskers) and cytokine data was presented as a median +/- interquartile range (IQR). An unpaired non-parametric Mann-Whitney U-test was used to compare cellular and cytokine data between preterm and term groups. A Spearman’s correlation was used to correlate gestational age and immune cell subsets. The data was graphically represented and statistically analysed using Graphpad prism v8 software (Graphpad Software Inc, California, USA). All tests performed were two-tailed and a p-value <0.05 was considered significant.

## Results

### Characteristics of Sample Cohort

Overall, 50 infants were recruited for this study with an even distribution of preterm and terms. The median gestation age for preterm infants was 32.6 (range: 30.4-34.1) weeks and 39.1 (range: 37-40) weeks for term infants. In the preterm group, 48% were male, whilst 68% were male in the term group. Additionally, all infants were born *via* normal vaginal delivery and no infants had presence of clinical chorioamnionitis (**Table 1**).

**Table 1:** Characteristics of preterm and term infants.

	Preterm N = 25	Term N = 25
**Gestational Age (wk)^1^**	32.6 (30.4-34.1)	39.1 (37-40)
**Sex^2^**	13 F, 12 M	8 F, 17 M
**Vaginal birth^3^**	25/25 (100)	25/25 (100)
**Clinical chorioamnionitis^3^**	0/25 (0)	0/25 (0)

^1^Data presented as median (range).

^2^F, female; M, Male.

^3^Data presented as n (%).

### Characterisation of Innate Immune Cells in Preterm and Term Infants

**Figure 1:**
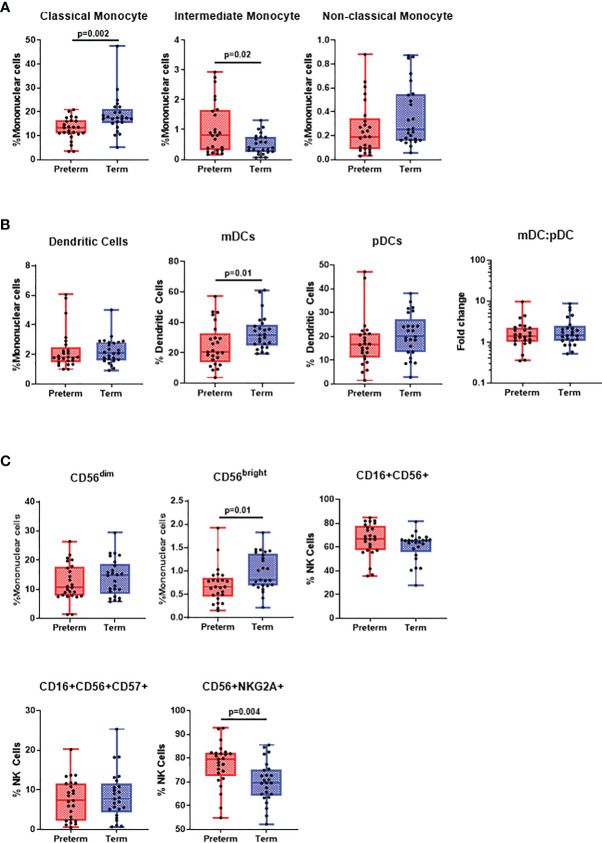
*Differences in innate cell populations in preterm and term infants*. Flow cytometry was performed on cord blood mononuclear cells from 25 preterm and 25 term infants. **(A)** Classical monocytes, intermediate monocytes and non-classical monocytes were expressed as % of mononuclear cells. All data was presented as a median +/- interquartile range (IQR) with min-max whiskers. **(B)** Dendritic cells were expressed as % of mononuclear cells, while myeloid and plasmacytoid dendritic cells were expressed as % of dendritic cells. **(C)** CD56^dim^ and CD56^bright^ NK cells were expressed as % of mononuclear cells whilst CD16+, CD16+CD57+ and NKG2A+ NK cells were expressed as % of total NK cells. A Mann-Whitney U-test was performed for all analysis and a p<0.05 was considered significant.

We examined innate immune profiles as diminished innate immunity may often result in skewed adaptive responses that lead to severe disease in preterm infants. Preterm infants exhibited a decreased frequency of CD14+CD16- classical monocytes (p=0.002) and an increased frequency of CD14+CD16+ intermediate monocytes (p=0.020) compared to term infants (**Figure 1A**). The frequency of CD14-CD16+ non-classical monocytes were similar between the groups. Preterm infants had a reduced myeloid dendritic cell (mDCs) frequency (p=0.010). Total dendritic cell (DC) and plasmacytoid dendritic cell (pDCs) frequencies were lower in preterm infants but this was not significant (**Figure 1B**). This trend is supported as we found a positive correlation between gestational age and pDC frequency in preterm infants (r=0.48, p=0.01; **Supplementary Figure 3B**). Additionally, the mDC to pDC ratio was no different between preterm and term infants (**Figure 1B**). Preterm infants had a reduced frequency of CD56^bright^ NK cells (p=0.010) and an increased frequency of inhibitory NKG2A+ NK cells (p=0.004), while other NK cell subsets, total CD56^dim^, activated (CD16+CD56+) and mature (CD16+CD56+CD57+) were similar between preterm and term infants (**Figure 1C**). Analyses of innate immune populations by gender revealed similar trends to these observations (**Supplementary Figure 4**).

### Characterisation of Adaptive Immune Cells in Preterm and Term Infants

**Figure 2:**
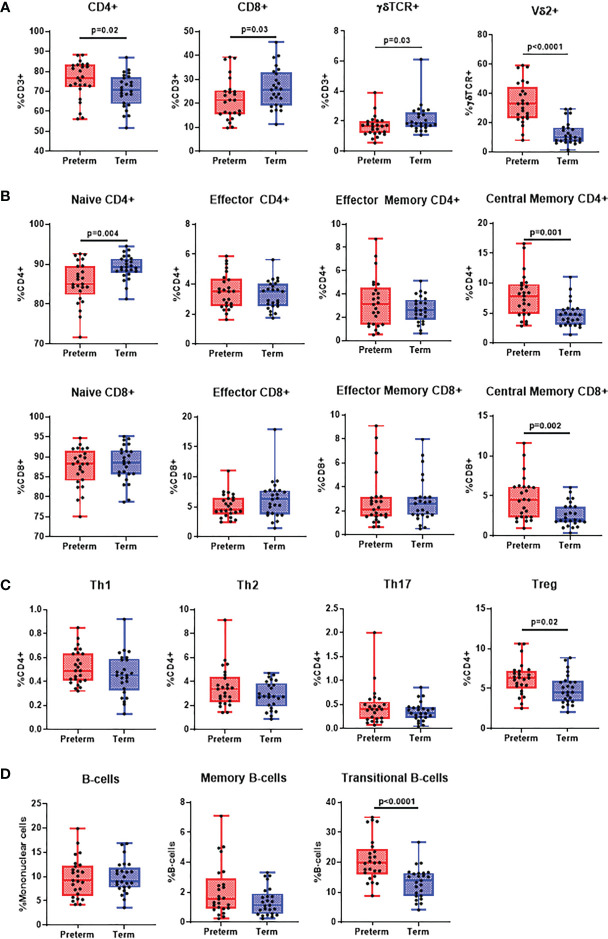
*Differences in adaptive immune cell populations in preterm and term infants.* Flow cytometry was performed on cord blood mononuclear cells from 25 preterm and 25 term infants. All data was presented as a median +/- IQR with min-max whiskers. **(A)** General T-cell subsets CD4+ CD8+ and γδ T-cells were expressed as % of CD3+ cells, whilst Vδ2+ was expressed as % of γδ T-cells. **(B)** Memory CD4+ and CD8+ T-cells were categorised into naïve, effector, central memory or effector memory and expressed as % of CD4+ or CD8+ T-cells. **(C)** CD4+ subsets were categorised as Th1, Th2, Th17 and Treg and expressed as % of CD4+ T-cells. **(D)** Total and memory B-cell subsets were expressed as % of mononuclear cells whilst IgD+ memory B-cells was expressed as % of memory B-cells and transitional B-cells were expressed as % of total B-cells. A Mann-Whitney U-test was performed for all analysis and a p<0.05 was considered significant.

Preterm infants had an increased proportion of CD4+ αβ T-cells (p=0.020), while CD8+ αβ T-cells were reduced (p=0.030). Interestingly, while total γδ T-cell proportions were reduced in preterm infants (p=0.030), γδ T-cells expressing Vδ2 were higher than in term infants (p<0.0001; **Figure 2A**). Memory populations within CD4+ and CD8+ T-cells were mostly similar except central memory (CM) CD4+ (p=0.001) and CD8+ T-cells (p=0.002) which were higher in preterm infants and naïve CD4+ T-cells (p=0.004) which were lower (**Figure 2B**). Treg frequencies were higher in preterm infants (p=0.020) while no differences were observed for Th1 (CD4+CXCR3+CCR4-), Th2 (CD4+CXCR3-CCR4+CCR6-) and Th17 (CD4+CXCR3-CCR4+CCR6+CD161+) cells (**Figure 2C**). Similarly, there were no differences in the frequency of B-cells and memory B-cells, although there was an increased frequency of transitional B-cells in preterm infants (p<0.0001; **Figure 2D**). Only CD4+ effector T cells (r=0.45, p=0.02) and Tregs (r=0.39, p=0.0.05) positively correlated with gestational age (**Supplementary Figures 5B, C**) in preterm infants. Comparisons of adaptive immune cell populations by gender also revealed similar trends to these data (**Supplementary Figure 6**).

### Cytokine, Chemokine, and Growth Factor Levels in Preterm and Term Infants

Cytokines, chemokines, and growth factors are crucial in influencing activation and proliferation of innate and adaptive immune cells to control and clear infection. Pro-inflammatory cytokines IL-1β (p=0.040), IL-6 (p=0.050) and IL-17A (p<0.0001) were lower in the serum from preterm infants whilst IL-15 (p=0.010) was higher compared to term infants. However, no differences in IL-2, IL-12, IFN-γ and TNF-α were observed. All anti-inflammatory cytokines (IL-1RA, IL-4, IL-5, IL-7, IL-9, IL-10, IL-13) were similar between preterm and term infant serum. Chemokines IL-8 (p=0.001), eotaxin (p=0.001), MIP-1α (p=0.0003) and MIP-1β (p=0.002) were all decreased in preterm infants whilst MCP-1 (p=0.0002) was elevated. RANTES and IP-10 were similar between the two groups. Lastly, growth factors were similar between preterm and term infants except for PDGF, which was elevated in term infants (p=0.001; **Table 2**). Comparisons for cytokines, chemokines and growth factors by gender revealed similar trends (**Supplementary Figure 7**).

**Table 2:** Plasma cytokine, chemokine, and growth factor levels in preterm and term infants*.

Pro-inflammatory cytokines	Preterm N = 25	Term N = 25	*p*
IL-1β	0.24 (0.17-0.35)	0.40 (0.22-0.84)	**0.04**
IL-2	0.45 (0.45-3.63)	0.45 (0.45-3.30)	0.83
IL-6	2.27 (1.59-3.07)	3.46 (2.12-10.60)	**0.05**
IL-12	13.89 (9.94021.42)	18.62 (11.11-25.34)	0.16
IL-15	24.97 (5.35-49.47)	4 (4-28.15)	**0.02**
IL-17A	7.82 (5.36-10.54)	13.98 (11.11-22.86)	**<0.0001**
IFN-γ	19.72 (15.94-26.17)	21.29 (15.69-28.75)	0.88
TNF-α	17.08 (12.32-22.58)	15.96 (11.32-22.38)	0.75
**Anti-inflammatory cytokines**			
IL-1RA	175.10 (120.2-250.5)	167.50 (119.3-214.2)	0.72
IL-4	1.30 (1.05-1.69)	1.26 (1.04-1.57)	0.79
IL-5	1.25 (1.25-4.96)	1.25 (1.25-3.06)	0.30
IL-7	7.73 (4.04-9.72)	5.96 (4.73-9.42)	0.72
IL-9	8.26 (6.59-10.78)	8.60 (7.61-12.11)	0.27
IL-10	1.66 (1.03-3.10)	1.86 (0.95-2.57)	0.85
IL-13	0.85 (0.52-1.23)	0.97 (0.53-1.22)	0.92
**Chemokines**			
IL-8	20.90 (11.61-41.05)	57.41 (34.68-214.4)	**0.001**
Eotaxin	2.69 (1.96-2.95)	3.51 (2.73-4.74)	**0.001**
MIP-1α	0.38 (0.17-0.90)	1.64 (0.84-3.69)	**0.0003**
MIP-1β	8.40 (4.63-11.89)	13.65 (10.00-26.64)	**0.003**
MCP-1	22.26 (15.93-28.71)	12.3 (10.37-19.20)	**0.0002**
RANTES	147.5 (124.1-186.7)	164.0 (144.3-208.0)	0.20
IP-10	72.25 (44.96-102.8)	73.96 (58.35-104.20)	0.37
**Growth Factors**			
G-CSF	64.84 (38.88-73.96)	52.65 (35.84-71.64)	0.16
FGF Basic	23.03 (5.80-37.72)	32.65 (16.74-44.19)	0.12
PDGF	789 (596-1391)	1437 (1064-2202)	**0.001**
VEGF	226.4 (60.56-359.20)	233.6 (167.0-397.9)	0.41

*All data are presented as median (IQR).

Bold values represent significantly different cytokines, chemokines and growth factors.

## Discussion

Comparative studies of immune profiles often involve adults, and to a lesser degree children. While many studies have compared aspects of preterm and term immune profiles, studies that have performed in-depth comparisons of both innate and adaptive immune profiles are limited. Our study provides a comprehensive insight into the immune landscape of infants born preterm versus those born full term. We identify significant differences in adaptive and innate immune cell composition between preterm and term infants, which may explain why preterm infants are more susceptible to severe infectious disease. Our data also reveals significant differences in serum cytokines and chemokines, particularly those that involved in pro-inflammatory immune responses.

We characterised the immune landscape of moderate preterm infants compared to term infants. Moderate preterm infants have an increased risk of severe infectious disease (9) and are therefore important groups for investigation. A recent paper by Olin et al. also compared preterm versus term infants but focused on infants <30wGA (10). While this is important, preterm infants born <30 wGA represent a smaller proportion of preterm infants than those born >30 wGA. The data presented in this paper provides insights into the immunological basis of susceptibility to disease in moderate preterm infants and possible therapeutic interventions (10).

Studies often report contradictory findings when comparing classical monocyte frequencies in preterm and term infants (5, 11). Classical monocytes in our study were reduced in preterm infants. A balanced pro-inflammatory response is crucial to controlling infectious pathogens (12). Therefore, given the importance of classical monocytes in phagocytosis, producing early pro-inflammatory cytokines (IL-1β, IL-6, and TNF-α) and chemokines (IL-8, eotaxin, macrophage inflammatory proteins) and T-cell activation, their reduction in preterm infants is likely detrimental during infection (13). We found that intermediate monocytes were increased in preterm infants. One study has related this to the immaturity of the innate immune system in preterm infants showing a much lower frequency of intermediate monocytes in term infants, which was further decreased in adults (14).

DCs are crucial in antigen presentation and type-1 interferon production and data comparing DCs subsets in preterm and term infants is limited. Our data suggests a reduced frequency of pDCs in preterm infants. This is skewed to non-significance by two outliers in the preterm group. A lower pDC frequency in preterm infants may explain their increased susceptibility to severe viral disease as pDCs are crucial for protection through the production of IFN-α (15). In our study, we observed a reduced frequency of mDCs in preterm infants compared to term infants. This is contrary to another study that had found an increased mDC frequency in preterms (11). Differences in cohort ethnicity, gating strategy or antibody cocktails be responsible for this. A reduced mDC population may not be surprising in the context of increased susceptibility to infectious disease as these cells produce cytokines IL-1β, IL-6, IL-12, and IL-23 following toll-like receptor stimulation of which IL-1β and IL-6 were reduced in preterm compared to term infants in our study (16, 17). This may also impact cross-talk between DCs and NK cells as secretion of IL-12 by mDCs activates NK cells to promote efficient viral immunity and clear differences were observed in NK cell populations (18).

NK cells are vital to anti-viral immunity through the production of IFN-γ and cytotoxic proteins. Lower frequencies of NK cells have been previously reported in preterms, however, this study did not compare differences in NK cell subsets (19). We found CD56^bright^ NK cells to be decreased in preterm infants whilst inhibitory NKG2A+ NK cells were increased. CD56^bright^ NK cells are efficient IFN-γ producers and NKG2A+ is an inhibitory marker to suppress cytokine secretion and cytotoxic function (20, 21). Accordingly, our data suggests that the anti-viral function of NK cells in preterm infants may be diminished compared to term infants, providing an explanation for their increased susceptibility to viral infection.

We found that preterm infants exhibited an increased CD4+ T-cell frequency and a reduction in CD8+T-cells compared with term infants. CD8+ T-cells are essential to viral clearance during infection and neonatal CD8+ T-cells are reported to have diminished functionality compared to adults (22, 23). Therefore, it is possible that the reduced numbers of CD8+ T cells we observed in preterm infants compared with term infants might contribute to their increased susceptibility as a result of reduced cytotoxicity (22). Memory subsets were mostly similar except CM CD4+ and CD8+ T-cells which were increased in preterm infants. This in part may be due to the increased memory T-cell activation that is associated with preterm birth (24). An overall reduction of γδ T-cells was seen in preterm infants, although, the frequency of γδ T-cell expressing the variable region 2 (Vδ2), which have potent anti-microbial activities, was increased. A similar result was found by Dimova et al. γδ+Vδ2+ T-cells, however this study did not find differences in total γδ T-cells between preterm and term infants, unlike our study (25). While γδ T-cells can be protective in bacterial and viral infection through their cytolytic proteins and pro-inflammatory cytokine production (26), their effectiveness in preterm infants is unclear. One study found that γδ T-cells in preterm infants have a reduced ability to produce pro-inflammatory cytokines (27). However, another study showed preterm infants as early as 20 wGA can mount effective γδ T-cell responses to cytomegalovirus *in utero* (28). Another major function of γδ T-cells is to induce the maturation of DCs. This in turn leads to differentiation of CD4+ T-cells into Th1 phenotypes to promote pro-inflammatory responses, especially during viral infection. Therefore, reduced γδ T-cell function coupled with their overall lower frequency may contribute to increased susceptibility to viral infection in preterm infants through reduced DC maturation (29).

No differences were observed in Th1, Th2 and Th17 frequencies. This was unexpected as preterm infants are thought to have increased Th2 skewing during infection leading to more severe disease (30). Evidence of this stems from innate immaturity influencing downstream adaptive immune responses and more definitive studies are required to compare Th1 responses between preterm and term infants. Our data revealing increased Treg cells in preterm infants compared to term infants supports data from a previous study (5) and although Treg cells control pro-inflammatory immune responses, it is unclear whether their higher abundance is detrimental in RSV infection. Some evidence suggests that an impaired capacity of Tregs to suppress inflammation is associated with severe RSV disease and is more common in preterm infants (31). In contrast, an abundance of Tregs leads to prolonged carriage of *Streptococcus pneumoniae* and disrupts pneumococcal clearance (32). B-cell phenotypes in preterm and term infants were relatively similar except for the frequencies of transitional B-cells. These cells tend to decrease with age as they are precursors to mature B-cells, therefore, a higher frequency in preterm infants is not unexpected (33). They also have the capacity to produce IL-10 and are associated with suppressing Th1 or Th17 differentiation, thereby, reducing pro-inflammatory responses (34). This is in line with the reduced pro-inflammatory responses associated with susceptibility to severe viral disease (35).

Serum cytokines and chemokines were mostly reduced in preterm infants. Levels of IL-1β, IL-6, IL-8, IL-17A, eotaxin, MIP-1α and MIP-1β were reduced whilst IL-15 and MCP-1 were increased. This suggests an overall reduced pro-inflammatory phenotype in preterm infants. The increased levels of MCP-1 in preterm infants could be related to intrauterine inflammation which is associated with preterm birth (36). While elevated levels of cytokines and chemokines can be associated with disease (37), these are also necessary for immune cell activation. For example, increased IL-8 production in infant T-cells leads to greater activation of neutrophils and γδ T-cells during infection (38). However, whether these baseline serum levels influence the cellular profile, capacity of cellular activation and their contribution to infectious susceptibility in preterm infants is unclear.

A major limitation is the lack of histological investigation of chorioamnionitis in this cohort, which is known to influence the immune response (39). This is not routinely done in Vietnam and so this data was not available. Another limitation to this study is the relatively small sample size. This is evident in our comparison of pDCs where the data trends towards a significant reduction in preterm infants but is skewed due to outliers. However, we have undertaken detailed immune profiling in this cohort to provide an overall immunological landscape between preterm and term infants. Additionally, our cohort data was not influenced by mode of delivery or the development of early onset of infection, which are factors known to influence immune profiles (40, 41). Intracellular staining to better categorise T-cell profiles may be more informative than surface staining of chemokine receptors to identify different T helper subsets.

## Conclusion

Characterisation of immune landscape in preterm and term infants has identified several important differences, suggesting a reduced capacity for pro-inflammatory immune responses in premature infants. This work represents a major advance in our understanding of immune cell composition of infants born moderate preterm and helps to explain why they are more susceptible to severe infectious disease. Studies of this nature could lead to identifying methods to attenuate the burden of disease in this highly vulnerable group.

## Data Availability Statement

The raw data supporting the conclusions of this article will be made available by the authors, without undue reservation.

## Ethics Statement

This study was approved by the Pasteur Institute Ho Chi Minh City Ethics Committee (Ethics approval: 213/QD-PAS) and RCH Human Research Ethics Committee (HREC; 56904). The patients/participants provided their written informed consent to participate in this study.

## Author Contributions

JA collected the data, performed the analyses and wrote the original draft. CT and LT coordinated the clinical aspects of this study of samples, contributed to study design and revised the manuscript. VD and PThanh coordinated samples and isolation of CBMCs and revised the manuscript. BN, DT, and PTrinh performed patient enrolment, sample collection and revised the manuscript. TN and NT contributed to the study design and revised the manuscript. CH, KM, and DP critically revised the manuscript and contributed to the interpretation of data. LD and PL conceived the study, provided major input into the manuscript revision and analysis. All authors approved the final manuscript as submitted and agree to be accountable for all aspects of the work.

## Funding

This work was funded by the Murdoch Children’s Research Institute Theme Research Grant. JA is supported by an Australian Postgraduate Award Scholarship. DP is supported by CSL Centenary Fellowship. PL is a recipient of an Australian National Health and Medical Research Council Career Development Fellowship (GNT1146198).

## Conflict of Interest

The authors declare that the research was conducted in the absence of any commercial or financial relationships that could be construed as a potential conflict of interest.

## Publisher’s Note

All claims expressed in this article are solely those of the authors and do not necessarily represent those of their affiliated organizations, or those of the publisher, the editors and the reviewers. Any product that may be evaluated in this article, or claim that may be made by its manufacturer, is not guaranteed or endorsed by the publisher.

## Supplementary Material

Supplementary Table 1 Flow cytometry antibody cocktails for cord blood mononuclear cell phenotyping and the suppliers of each antibody.

Supplementary Figure 1 Gating strategy for innate cells and B-cells from cord blood mononuclear cells. Innate cells were identified by CD3-CD19- expression on live single mononuclear cells. Innate cells were further categorised into NK cells (CD56+CD14-HLA-DR-) and HLA-DR+CD14+ cells. NK cells were further categorised into CD56^dim^ and CD56^bright^. From CD56^dim^ NK cells CD16+ and CD16+CD57+ cells were gated. NKG2A+ expression was gated from total NK cells. From the HLA-DR+CD14+ fraction, cells were divided into classical monocytes (CD14+CD16-), intermediate monocytes (CD14+CD16+), non-classical monocytes (CD14-CD16+) and dendritic cells (HLA-DR+CD14-CD16-). Dendritic cells were further categorised into myeloid dendritic cells (CD11c+) and plasmacytoid dendritic cells (CD123+). B-cells were identified by CD3-CD19+ expression on live single leukocytes. B-cells that expressed CD27 were considered memory B-cells and B-cells expressing CD24^hi^CD38^hi^ phenotypes were transitional B-cells.

Supplementary Figure 2 Gating strategy for T-cell subsets from cord blood mononuclear cells. T-cells were identified as CD3+ from live single lymphocytes. T-cells were further categorised into CD4+, CD8+, γδTCR+ and γδTCR+Vδ2+ T-cells. CD4+ and CD8+ T-cells were classified as naïve, effector, effector memory (EM) or central memory (CM) based on CCR7 and CD45RA expression. From the CD4+ T-cells we further identified their subsets. CXCR3+ cells were considered Th1, CXCR3-CCR4+CCR6- cells were considered Th2, CXCR3-CCR4+CCR6+CD161+ cells were considered Th17 and CD25^hi^CD127^lo^ cells were considered Tregs.

Supplementary Figure 3 Correlation between gestational age and innate cell subsets. **(A)** Monocyte subsets. **(B)** Dendritic cells and dendritic cell subsets. **(C)** NK cells and NK cell subsets. A spearman’s correlation was used to correlate gestational age and immune cell frequencies. A p<0.05 was considered significant.

Supplementary Figure 4 Comparison of innate immune cell populations by gender in preterm and term infants. **(A)** comparison between male preterm and term infants. **(B)** comparison between female preterm and term infants. A Mann Whitney U-test was used to compared cell frequencies and a p<0.05 was considered significant.

Supplementary Figure 5 Correlation between gestational age and T-cell and B-cell subsets. **(A)** General T-cell populations **(B)** Memory CD4+ and CD8+ T-cells. **(C)** CD4+ T-cell subsets. **(D)** B-cells and B-cell subsets. A spearman’s correlation was used to correlate gestational age and immune cell frequencies. A p<0.05 was considered significant.

Supplementary Figure 6 Comparison of adaptive immune cell populations by gender in preterm and term infants. **(A)** comparison between male preterm and term infants. **(B)** comparison between female preterm and term infants. A Mann Whitney U-test was used to compared cell frequencies and a p<0.05 was considered significant.

Supplementary Figure 7 Comparison of cytokines, chemokines and growth factors by gender in preterm and term infants. **(A)** comparison between male preterm and term infants. **(B)** comparison between female preterm and term infants. A Mann Whitney U-test was used to compared concentrations and a p<0.05 was considered significant.
