# Supplementary material for: Immune Profiling of Cord Blood From Preterm and Term Infants Reveals Distinct Differences in Pro-Inflammatory Responses
Source: Front Immunol. 2021 Nov 1;12:777927. doi: 10.3389/fimmu.2021.777927 (PMC8591285; doi:10.3389/fimmu.2021.777927)
Supplement: Supplementary Table 1 — Flow cytometry antibody cocktails for cord blood mononuclear cell phenotyping and the suppliers of each antibody. [file Table_1.docx]

| Antibody Cocktail 1 | Supplier | Antibody Cocktail 2 | Supplier |
| --- | --- | --- | --- |
| CXCR3-APC | BD Bioscience, San Diego, CA, USA | CD3-PerCPcy5.5 | BD Bioscience, San Diego, CA, USA |
| CCR6-BUV496 | BD Bioscience, San Diego, CA, USA | CD16-BV605 | BD Bioscience, San Diego, CA, USA |
| CCR4-BV605 | BioLegend, San Diego, USA | CD24-BV711 | BioLegend, San Diego, USA |
| γδTCR-FITC | BD Bioscience, San Diego, CA, USA | HLA-DR-APC | BD Bioscience, San Diego, CA, USA |
| CD127-APC-R700 | BioLegend, San Diego, USA | CD11c-FITC | BioLegend, San Diego, USA |
| CD25-PE-CF594 | BD Bioscience, San Diego, CA, USA | CD20-BV421 | BioLegend, San Diego, USA |
| Vδ2-PE | BD Bioscience, San Diego, CA, USA | CD56-BV510 | BioLegend, San Diego, USA |
| CD161-PEvio770 | Miltenyi Biotec, New South Wales, Australia | CD14-BUV805 | BD Bioscience, San Diego, CA, USA |
| CD3-BUV395 | BD Bioscience, San Diego, CA, USA | NKG2A-PE | Beckman Coulter, California, USA. |
| CD4-BV421 | BD Bioscience, San Diego, CA, USA | CD123-PEcy7 | BD Bioscience, San Diego, CA, USA |
| CD8-BUV805 | BD Bioscience, San Diego, CA, USA | CD27-BUV737 | BD Bioscience, San Diego, CA, USA |
| CCR7-BV785 | BioLegend, San Diego, USA | IgD-BUV395 | BD Bioscience, San Diego, CA, USA |
| CD45RA-Percp/Cy5.5 | BD Bioscience, San Diego, CA, USA | CD19-BV785 | BioLegend, San Diego, USA |
| Zombie NIR | BioLegend, San Diego, USA | CD57-PE-CF594 | BD Bioscience, San Diego, CA, USA |
|  |  | CD38-BUV496 | BD Bioscience, San Diego, CA, USA |
|  |  | Zombie NIR | BioLegend, San Diego, USA |
