# Supplementary figures and images for: Immune Profiling of Cord Blood From Preterm and Term Infants Reveals Distinct Differences in Pro-Inflammatory Responses
Source: Front Immunol. 2021 Nov 1;12:777927. doi: 10.3389/fimmu.2021.777927 (PMC8591285; doi:10.3389/fimmu.2021.777927)

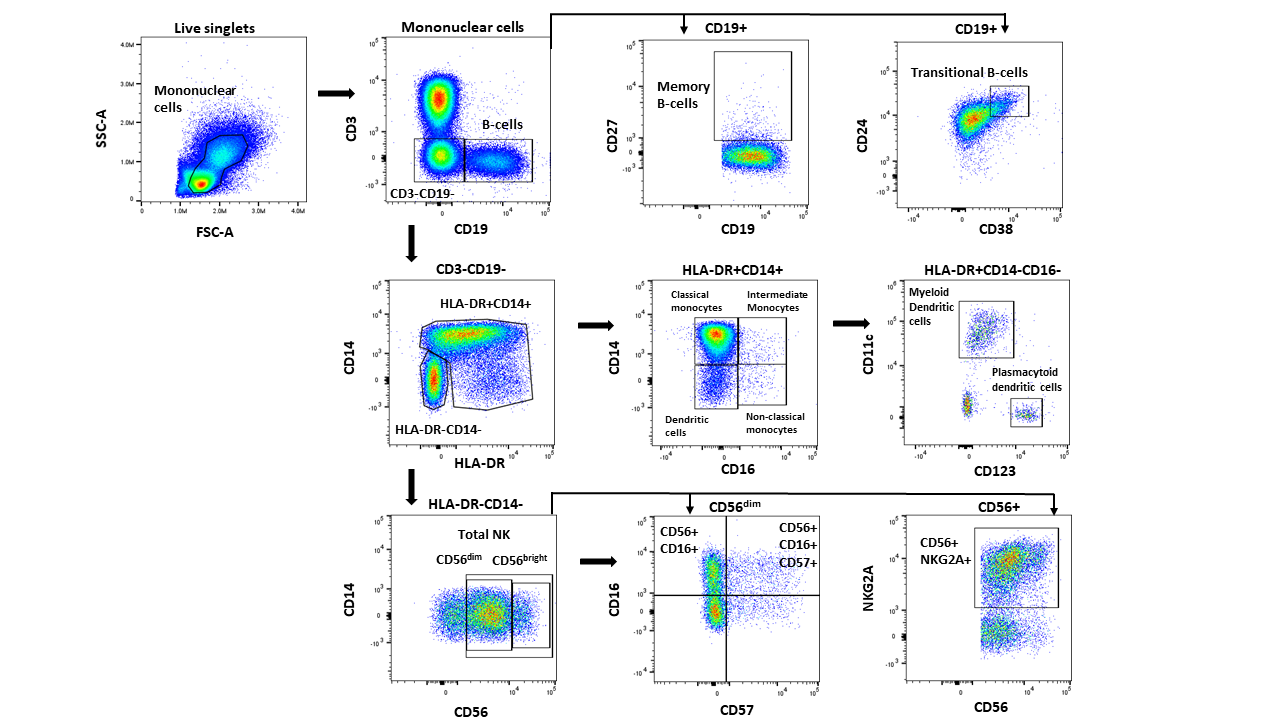

Supplement: Supplementary Figure 1 — Gating strategy for innate cells and B-cells from cord blood mononuclear cells. Innate cells were identified by CD3-CD19- expression on live single mononuclear cells. Innate cells were further categorised into NK cells (CD56+CD14-HLA-DR-) and HLA-DR+CD14+ cells. NK cells were further categorised into CD56dim and CD56bright. From CD56dim NK cells CD16+ and CD16+CD57+ cells were gated. NKG2A+ expression was gated from total NK cells. From the HLA-DR+CD14+ fraction, cells were divided into classical monocytes (CD14+CD16-), intermediate monocytes (CD14+CD16+), non-classical monocytes (CD14-CD16+) and dendritic cells (HLA-DR+CD14-CD16-). Dendritic cells were further categorised into myeloid dendritic cells (CD11c+) and plasmacytoid dendritic cells (CD123+). B-cells were identified by CD3-CD19+ expression on live single leukocytes. B-cells that expressed CD27 were considered memory B-cells and B-cells expressing CD24hiCD38hi phenotypes were transitional B-cells. [file Image_1.tif]

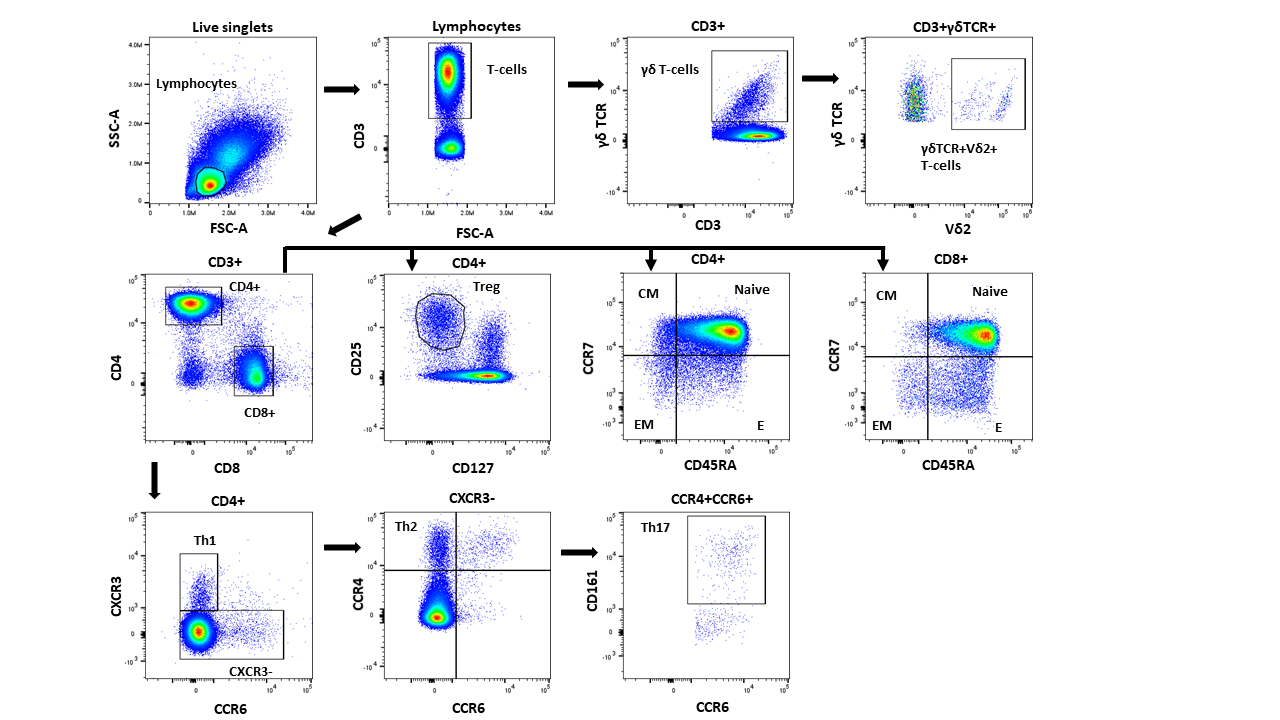

Supplement: Supplementary Figure 2 — Gating strategy for T-cell subsets from cord blood mononuclear cells. T-cells were identified as CD3+ from live single lymphocytes. T-cells were further categorised into CD4+, CD8+, γδTCR+ and γδTCR+Vδ2+ T-cells. CD4+ and CD8+ T-cells were classified as naïve, effector, effector memory (EM) or central memory (CM) based on CCR7 and CD45RA expression. From the CD4+ T-cells we further identified their subsets. CXCR3+ cells were considered Th1, CXCR3-CCR4+CCR6- cells were considered Th2, CXCR3-CCR4+CCR6+CD161+ cells were considered Th17 and CD25hiCD127lo cells were considered Tregs. [file Image_2.tif]

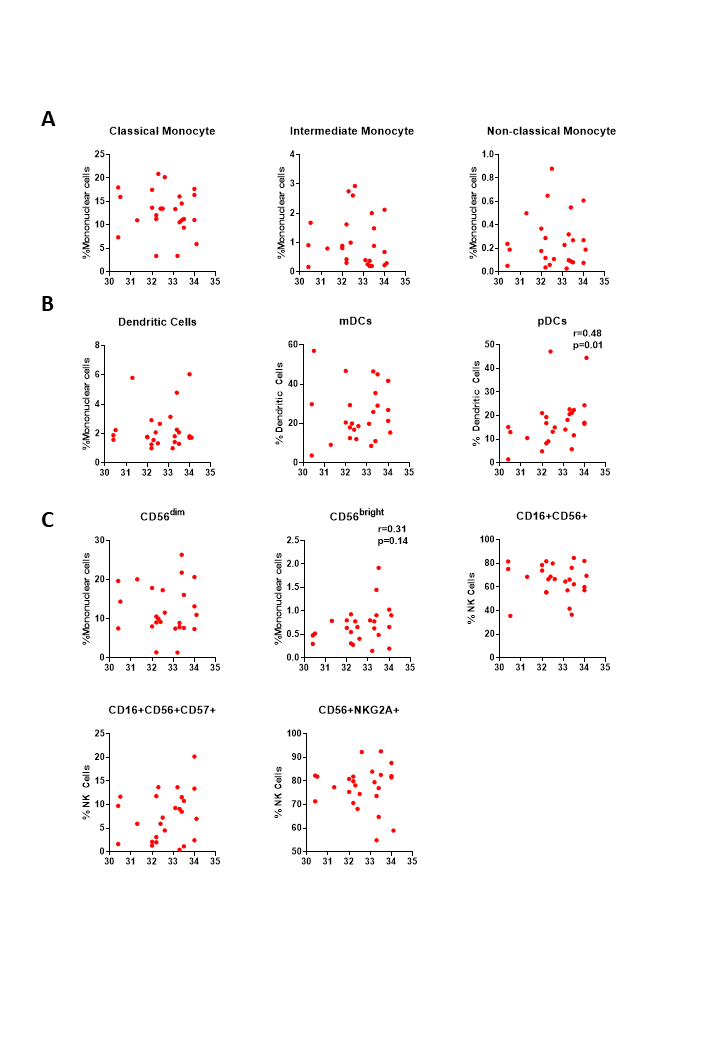

Supplement: Supplementary Figure 3 — Correlation between gestational age and innate cell subsets. (A) Monocyte subsets. (B) Dendritic cells and dendritic cell subsets. (C) NK cells and NK cell subsets. A spearman’s correlation was used to correlate gestational age and immune cell frequencies. A p<0.05 was considered significant. [file Image_3.tif]

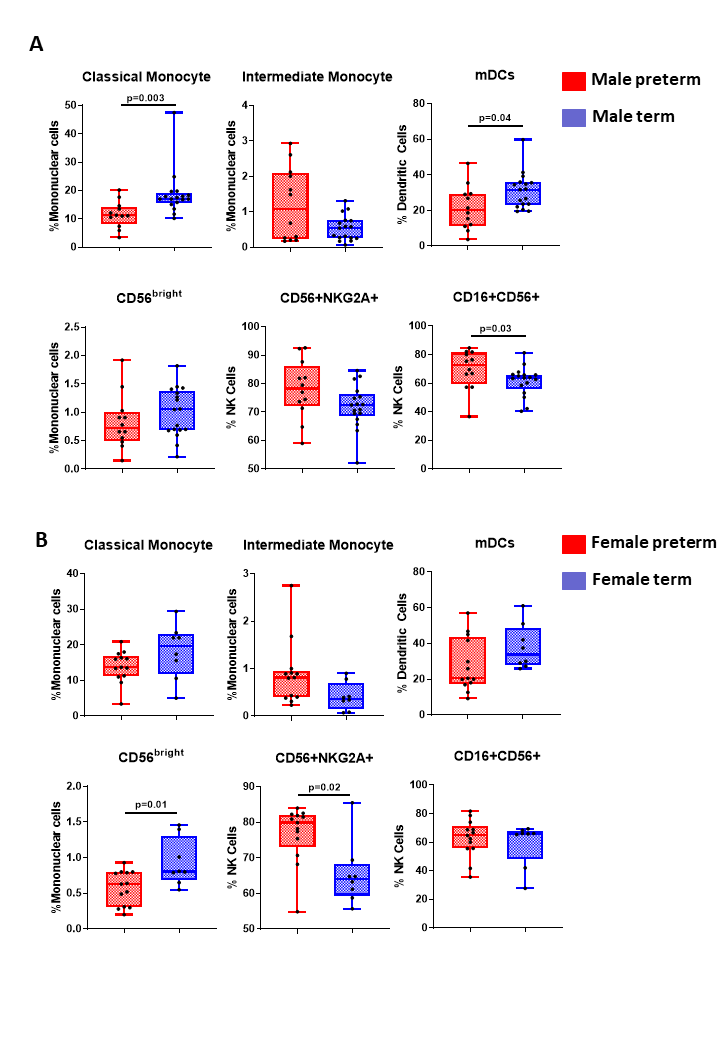

Supplement: Supplementary Figure 4 — Comparison of innate immune cell populations by gender in preterm and term infants. (A) comparison between male preterm and term infants. (B) comparison between female preterm and term infants. A Mann Whitney U-test was used to compared cell frequencies and a p<0.05 was considered significant. [file Image_4.tif]

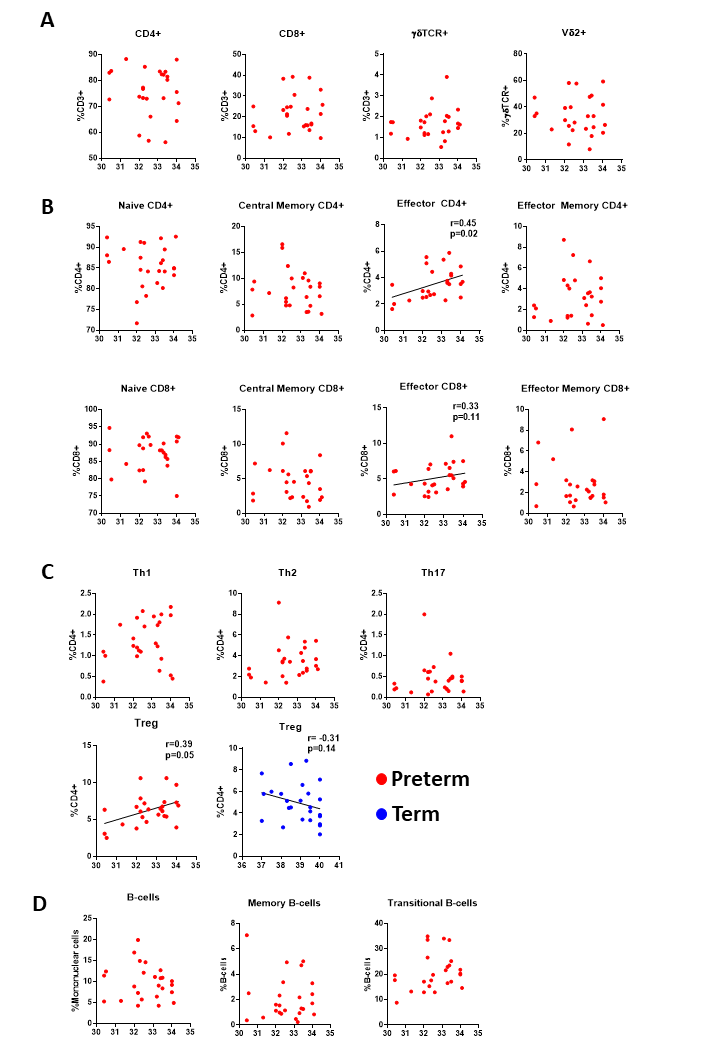

Supplement: Supplementary Figure 5 — Correlation between gestational age and T-cell and B-cell subsets. (A) General T-cell populations (B) Memory CD4+ and CD8+ T-cells. (C) CD4+ T-cell subsets. (D) B-cells and B-cell subsets. A spearman’s correlation was used to correlate gestational age and immune cell frequencies. A p<0.05 was considered significant. [file Image_5.tif]

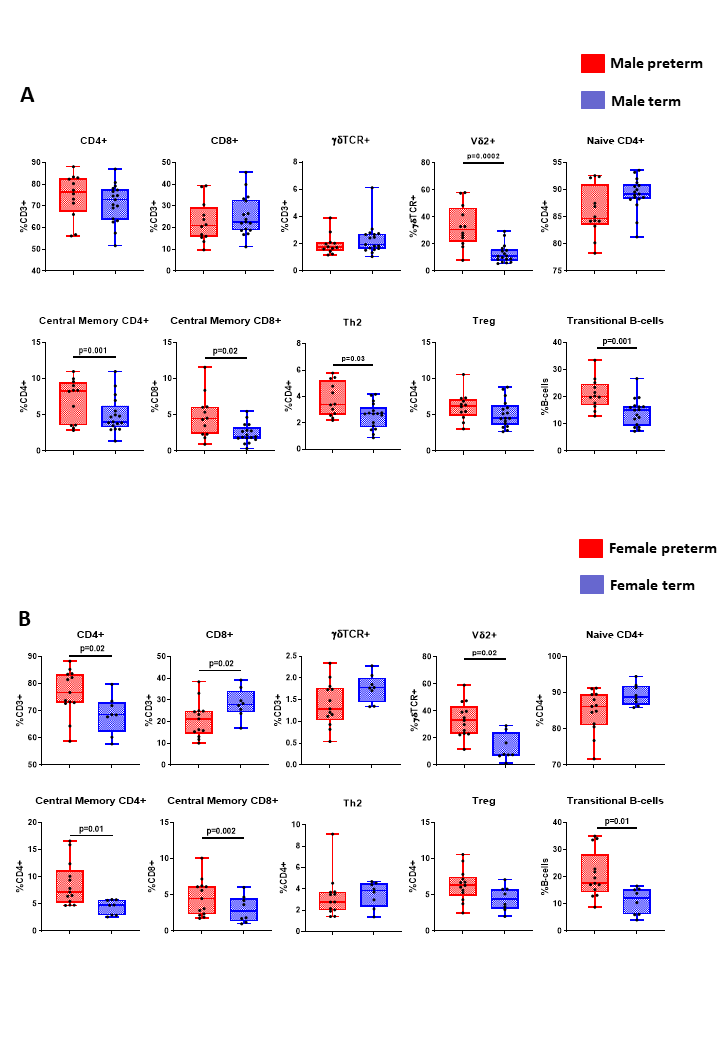

Supplement: Supplementary Figure 6 — Comparison of adaptive immune cell populations by gender in preterm and term infants. (A) comparison between male preterm and term infants. (B) comparison between female preterm and term infants. A Mann Whitney U-test was used to compared cell frequencies and a p<0.05 was considered significant. [file Image_6.tif]

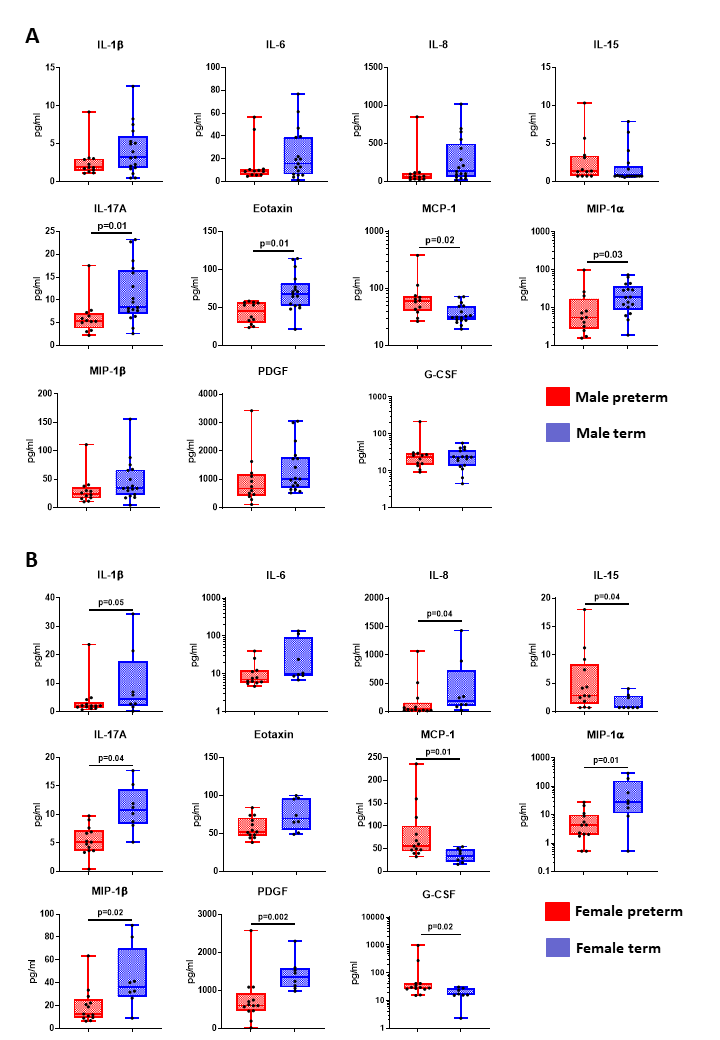

Supplement: Supplementary Figure 7 — Comparison of cytokines, chemokines and growth factors by gender in preterm and term infants. (A) comparison between male preterm and term infants. (B) comparison between female preterm and term infants. A Mann Whitney U-test was used to compared concentrations and a p<0.05 was considered significant. [file Image_7.tif]
